# A microarray data-based semi-kinetic method for predicting quantitative dynamics of genetic networks

**DOI:** 10.1186/1471-2105-6-299

**Published:** 2005-12-13

**Authors:** Katsuyuki Yugi, Yoichi Nakayama, Shigen Kojima, Tomoya Kitayama, Masaru Tomita

**Affiliations:** 1 Institute for Advanced Biosciences, Keio University, Tsuruoka, 997-0035, Japan

## Abstract

**Background:**

Elucidating the dynamic behaviour of genetic regulatory networks is one of the most significant challenges in systems biology. However, conventional quantitative predictions have been limited to small networks because publicly available transcriptome data has not been extensively applied to dynamic simulation.

**Results:**

We present a microarray data-based semi-kinetic (MASK) method which facilitates the prediction of regulatory dynamics of genetic networks composed of recurrently appearing network motifs with reasonable accuracy. The MASK method allows the determination of model parameters representing the contribution of regulators to transcription rate from time-series microarray data. Using a virtual regulatory network and a *Saccharomyces cerevisiae *ribosomal protein gene module, we confirmed that a MASK model can predict expression profiles for various conditions as accurately as a conventional kinetic model.

**Conclusion:**

We have demonstrated the MASK method for the construction of dynamic simulation models of genetic networks from time-series microarray data, initial mRNA copy number and first-order degradation constants of mRNA. The quantitative accuracy of the MASK models has been confirmed, and the results indicated that this method enables the prediction of quantitative dynamics in genetic networks composed of commonly used network motifs, which cover considerable fraction of the whole network.

## Background

With the advent of high-throughput biotechnologies in the last decade of the 20th century, enormous amounts of data have been generated on intracellular molecules [1-5]. The ongoing accumulation of such large-scale information presents a significant challenge to the scientific community: Namely, to understand the cell-wide molecular network as a living system [6-8]. In particular, modelling the behaviour of genetic regulatory networks has been one of the most significant milestones in systems biology [9-12]. In many previous studies, the dynamic behaviours of genetic networks were quantitatively predicted and analyzed in terms of non-linear ordinary differential equations based on reaction kinetics [13-15]. However, because it is arduous to obtain a complete set of sufficiently accurate kinetic properties of molecular interactions, the application of this method is limited to small regulatory networks, such as the tryptophan operon [15] and the lysis / lysogeny circuit of the bacteriophage lambda[13,14].

Recently, there have been a few attempts to construct mathematical models of gene expression from large-scale data sets. These include generating reproductions of time series microarray data by 'time translation matrix'[16], a parameterization of the *E. coli *SOS module model using green fluorescent protein (GFP) reporter plasmids[17], a qualitative simulation method based on piecewise linear differential equations[18] and network model inferences from microarray data using a dynamic Bayesian network[19] or a system identification technique[20]. However, each of these approaches has limitations. Time translation matrix models are incapable of explaining dynamic gene expression patterns beyond the actual training data set, i.e. the expression profiles used to generate the matrix models themselves. The usefulness of the GFP approach is restricted to groups of genes regulated by one regulator. By definition, qualitative simulation is not capable of predicting quantitative dynamics. Finally, it is not clear how kinetic models that are inferred via a dynamic Bayesian network or a system identification method can generate accurate, quantitative predictions even though they could infer regulatory connections of genetic networks. Hence, the establishment of dynamic simulation methods for large-scale genetic regulatory networks remains a challenging problem in systems biology.

Here, we present a microarray data-based semi-kinetic (MASK) method for dynamic simulation of genetic regulatory networks composed of common network motifs. The quantitative accuracy of the MASK method was validated using a virtual genetic network described in a previous study[21], as well as genetic module of *Saccharomyces cerevisiae *inferred from expression profiles and genome-wide location analysis data[22]. The virtual genetic network and the yeast network were employed to test the applicability of the MASK method to the frequently appearing network motifs: Single input motifs (SIMs) and multi-input motifs (MIMs), respectively. The yeast genetic module model is composed of 13 ribosomal protein (RP) genes regulated by Fhl1, Gal4 and Rap1. Recent experimental studies confirmed that Fhl1 and Rap1 bind upstream of yeast RP genes[4,23]. With respect to the galactose-sensitive regulator Gal4, it has been reported that galactose addition triggers a three- to five-fold increase in the mRNA levels of RP genes[9].

The MASK model accurately predicted not only the training data sets, but also the test data sets. A test data set comprises microarray data that is not used for model estimation. Since SIMs and MIMs appear recurrently in genetic regulatory networks[4,7], the results support the contention that the MASK method is applicable to a large fraction of the whole network.

## Results

### Method validation using a hypothetical model

**Figure 1:**
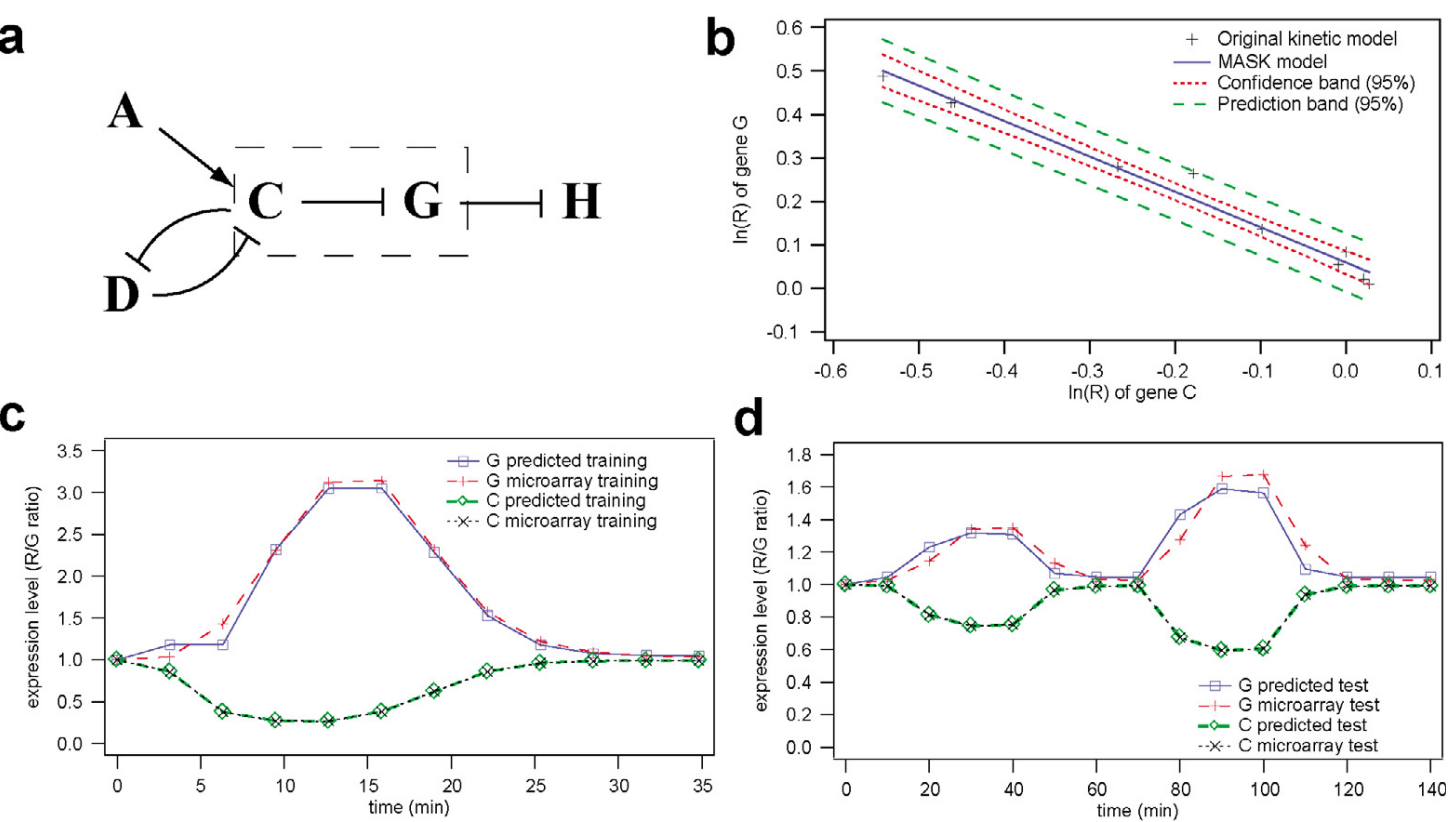
**Validation results of the MASK method using the virtual genetic network. **(a) Part of the virtual genetic network shown in Ref. [21]. The regulation of gene G by gene C was employed to compare the MASK method and conventional kinetic model. (b) A log-log scatter plot of the R values of gene C and G. (c) The training data used in estimating the MASK model parameters. (d) The test data used for the validation of the MASK model. Applied to the same gene C expression time series, the MASK model calculated the time course of gene G as accurately as the original kinetic model. The model parameters were not changed.

Initially, the accuracy of the MASK method was evaluated by comparison with a hypothetical regulatory network model based on conventional reaction kinetics[21]. The SBML format model of the network was imported from the supplementary website of Ref.[21]. In the MASK version model, the original rate equations for transcription of genes C and G (Figure 1a) were replaced with Eq. (1), which is shown in Methods. The dissociation constant of RNA polymerase to DNA was set at *K*_*s *_= 0.1 μM. The first-order decay constant of the original kinetic model was employed for the mRNA degradation of genes C and G. The R term (See Methods for detail) of the transcription rate equation for gene G was approximated by a power-law function of the R term of gene C, as shown in Eq. (2). Translation and dimerization of C in the original kinetic model were abstracted using a power-law function. A computer simulation was performed to obtain a training data set to estimate the coefficients of Eq. (2). These coefficients define the quantitative regulatory relationship between genes C and G. Calculated time courses of mRNA abundance were normalized to the initial copy numbers of each mRNA. We employed these normalized transcript time courses (12 time points, sampling interval = 3 minutes) as an alternative to time-series microarray data. The coefficients of Eq. (2) for genes C and G were estimated by regression analysis of these 'virtual microarray data' after correcting for time delay, estimated by the local clustering method[24].

The R values of gene C in the MASK version of the model were adjusted to reproduce the expression levels represented in the original data. This meant that the accuracy of the MASK method could be evaluated by mean relative error of the target gene, G, under the same conditions as the original kinetic model (See Additional Text 1 for the detailed procedure of R value adjustment). The mean relative errors of gene G generated by both the original kinetic model and the MASK model were calculated with respect to 12 points sampled from the time courses.

Next, the inferred MASK model was applied to predict an expression time course of gene G in another condition, without any parameter change. Note that this 'test data set' was not used to train the MASK model. As well as the previous comparison of the predicted time course of gene G to the training data set, the mean relative error of gene G was calculated under identical regulatory conditions.

Both in the training and the test data sets, the expression time course of gene G generated by the MASK model was similar to that of the original model (Figures 1c and 1d). A regression analysis using the training data sets (Figure 1b) revealed that the relationship between the R terms of genes C and G was *R*_*G *_= 1.15 *R*_*C *_- 0.81(*t*-190 min). The mean relative error over the time series was 3.95% in the training data set. This MASK model was then employed for the prediction of time evolutions in another condition to validate whether a MASK model can perform accurate computer simulations of various states, other than those represented by the training data sets. As mentioned above, the predicted time course of gene G mRNA was very similar for the MASK and the original kinetic models (Figure 1d). In the test data set, the mean relative error of the gene G time series expression profile was 4.19%.

### Application to a yeast genetic module using microarray data

**Figure 2:**
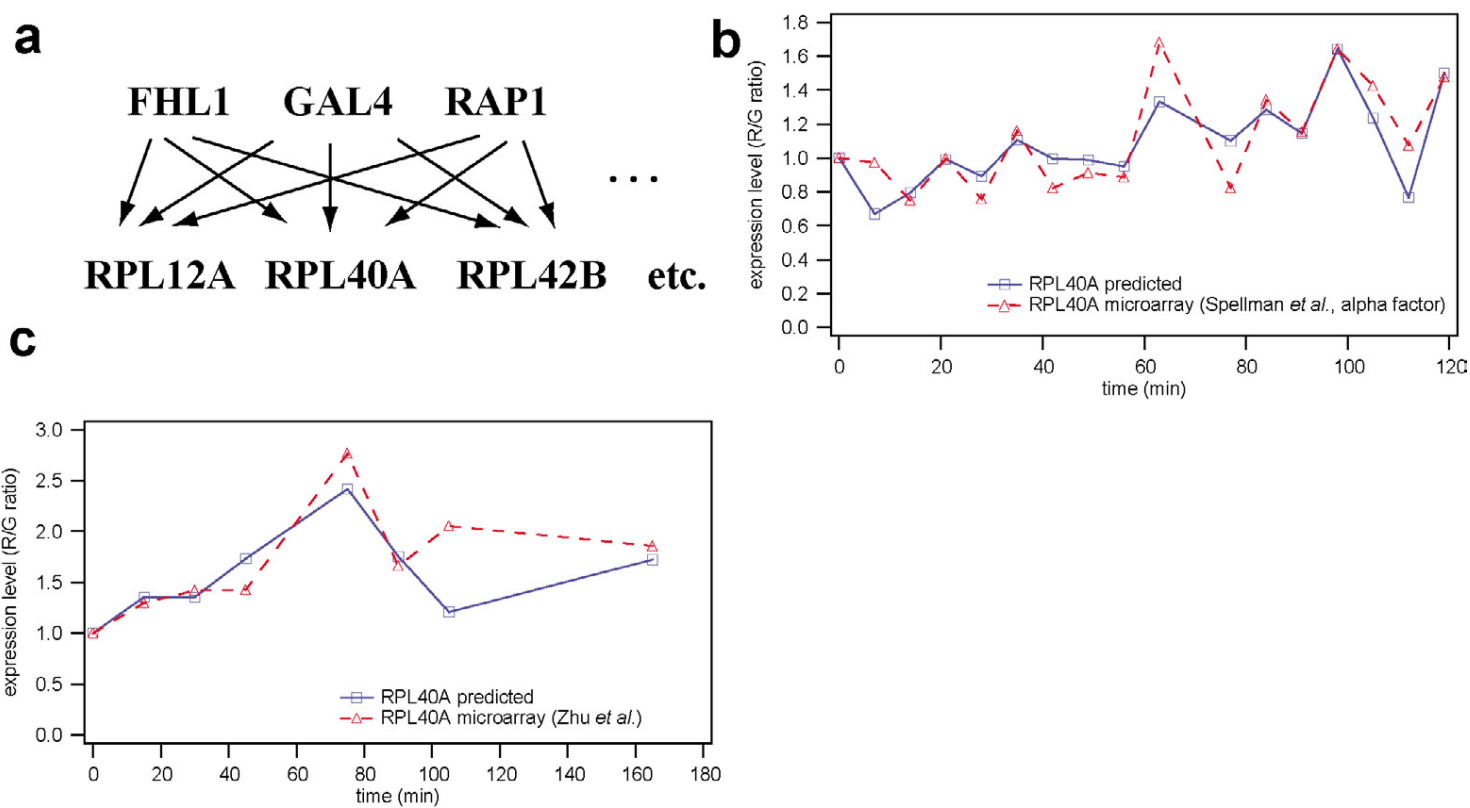
**Validation results of the MASK method using yeast RP genes. **(a) A genetic regulatory module of yeast RP genes described in Ref.[22]. Our model included 13 target genes of this module. (b) A comparison of the training microarray data [27] and a time course calculated by the MASK model. The mean relative error of RPL40A time series was 11.4%. (c) A comparison of the test data [28] and a calculated time series. The mean relative error of the RPL40A time series was 12.1%.

The MASK method was employed to predict expression profiles of a RP gene module of *Saccharomyces cerevisiae*. This genetic regulatory module is a MIM involving 13 target genes (RPL12A, RPL12B, RPL13A, RPL18A, RPL20B, RPL31A, RPL40A, RPL42B, RPP2A, RPS6B, RPS15, RPS23B, RPS24A and TEF1) which, on the basis of genome-wide location analysis data and expression profiling, are considered to be regulated by Fhl1, Gal4 and Rap1 (Figure 2a)[22]. The first order degradation constant (*k*_*deg*_) for each gene was obtained by comprehensive measurement of yeast mRNA degradation[25]. The concentration of RNA polymerase II holoenzyme was set at 10,000 molecules/cell based on the reported value from a proximal species, *Schizosaccharomyces pombe*[26]. The dissociation constant of RNA polymerase to DNA was set at *K*_*s *_= 0.1 μM. An expression profile of 'alpha-factor block'[27] was employed as the training data to estimate the model, owing to its abundant time points measured in a uniformly sampled time interval (18 time points with 7 min interval), which the local clustering method requires[24]. For the test data set, a microarray data set measured by Zhu *et al.*[28] was employed. The R values of the regulator genes were adjusted to reproduce the original expression levels of the regulators. Mean relative errors between the original microarray data and the simulation results were evaluated for each target gene.

Importantly, MASK modelling of the 13 *Saccharomyces cerevisiae *RP genes predicted similar temporal patterns of mRNA expression to both the training and test microarray data (Figure 2b and 2c). For the MASK model, the average mean relative error of the 13 target genes was 10.6% and 26.3% in comparison to the training data set ('alpha-factor block' by Spellman *et al.*[27]) and the test data set (Zhu *et al.*[28]), respectively. The mean relative error of each gene is shown in Table 1.

**Table 1:** Mean relative error of the RP genes.

	Training data[27]	Test data[28]
RPL12A	0.13	0.10
RPL12B	0.08	0.39
RPL13A	0.13	0.09
RPL18A	0.09	0.22
RPL20B	0.09	0.19
RPL31A	0.13	0.16
RPL40A	0.11	0.12
RPL42B	0.13	0.15
RPP2A	0.09	0.39
RPS15	0.10	0.38
RPS23B	0.09	0.22
RPS24A	0.11	0.34
RPS6B	0.08	0.35
TEF1	0.11	0.32

**Figure 3:**
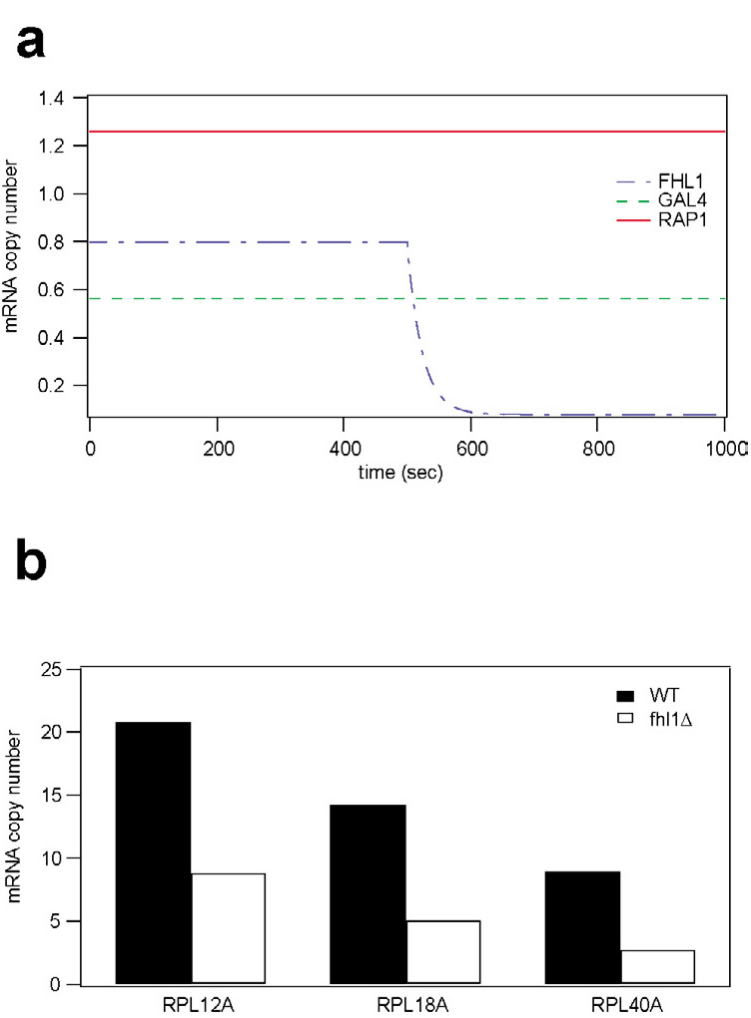
**A prediction of RP gene transcription in the fhl1Δ strain. **(a) The R value of FHL1 was changed to realize depletion of Fhl1 mRNA. The transcription rates of the other two regulators were unchanged. (b) Calculated RP mRNA levels in the wild-type (WT) and fhl1Δ strains. The 40–60% decrease is in agreement with a previous observation [29].

A simulation experiment was performed to determine whether the yeast RP gene module model is capable of predicting data other than microarray data. Specifically, we attempted to calculate transcript levels of the RP genes in the fhl1Δ strain, as these had been measured recently[29]. The deletion of fhl1 was represented by reducing the initial R value of Fhl1 from 1.0 to 0.1, thereby leading to depletion of Fhl1 mRNA (Figure 3a). Transcript levels of the RP genes were calculated at two time points; the initial steady-state level and another steady-state level after perturbation of Fhl1. The simulation results of the RP gene MASK model were consistent with recent experimental observations. It had been reported that transcript levels of two representative RP genes (RPL9A and RPL30) in the fhl1Δ strain were approximately 40–60% of those in the wild-type[29] and similar decreases in mRNA levels were predicted by the model (Figure 3b).

## Discussion

The MASK model reproduced the dynamic behaviour of the virtual network and the yeast genetic module with a sufficient degree of accuracy. Since the tested networks are examples of network motifs including a SIM and a MIM, the coverage of the MASK method is as wide as the frequency of the motifs. This successful example of a MIM regulated by Rap1, Fhl1 and Gal4 supports the contention that the MASK method is capable of representing synergistic effects between multiple co-regulators, since it has been reported that Rap1 binding at promoters is required for Fhl1 binding[30]. Furthermore, it has been theoretically demonstrated that the power-law equation can capture synergism of non-linear equations which are comprised of sums and products of elementary functions i.e. nearly all types of rate equations[31]. Presumably, the 3–5% errors observed between the mathematical models of the virtual genetic network are attributable to power-law approximation, microarray sampling interval and abstraction of translation by time delay. In particular, the error caused by power-law approximation may reflect the residuals of each data point from the regression line (Figure 1b). With respect to the RP gene module model, the mean relative errors between the prediction and the experimental time series are larger than those observed in the comparative study of the conventional kinetic and MASK models. We consider that the error increment is due to measurement errors in the microarray data, which does not occur in the 'virtual microarray data'. Examining replicated microarray data, such as that presented in Refs.[32,33], it is observed that on average, relative errors between the replicates are in the range 20–50% (data not shown), whereas the error between replicated GFP measurements is approximately 10%[17,34]. We did not employ replicated time series microarray data in this study, because no such data sets were found in the databases. Despite having a smaller measurement error than microarray data, the kinetic model based on GFP time series data still exhibited mean relative errors of 10–20%[17]. Thus, it is satisfactory that a microarray data-based model predicts gene expression dynamics with an error level of 10–30%. Moreover, microarray is significantly superior to GFP in terms of availability and comprehensiveness of data. This provides a rationale for immediate application of the MASK method to regulatory networks in as large a scale as microarray data allow.

Microarray data sets for training a MASK model should preferably have prominent variations in expression level and many time points within a short time interval. These desirable features of microarray data restrain the representation space of a MASK model. If the time series expression profile of both a regulator and its target gene are almost flat, it is obvious that their data points will not provide a significant regression line. For a meaningful regression, expression levels should be widely distributed by dynamic variations in transcription to provide sufficiently long confidence bands (e.g. Figure 1b), which guarantee broad representation space of the model. For microarray data, the resolution of time delay is restrained by time interval duration. This reflects the fact that the local clustering method quantifies the time delay by the number of time intervals. Therefore, short time intervals are preferable for quantifying time delays precisely.

Network architecture also restrains the application of the MASK method. The MASK method implicitly assumes that transcription rates of regulator genes are independent of those of target genes. Therefore, it is not appropriate in principle to employ the MASK method on particular genetic networks in which target genes largely influence the expression of their regulators – for example a pair of mutually regulating genes or a 'multi-component loop'[4]. Fortunately, this restriction does not substantially constrain the extensive application of the MASK method because only three multi-component loops have so far been identified in a yeast genome-wide location analysis[4].

As a consequence, it is plausible that there will be a drastic reduction in the requirement for detailed kinetic data given that all yeast genes in SIMs and MIMs, with the exception of regulators, could be modelled without kinetic data. Thus, the MASK method facilitates the prediction of quantitative, dynamic behaviour of gene networks with sufficient accuracy.

## Conclusion

We have demonstrated a novel method for the construction of dynamic simulation models of gene networks from time-series microarray data, initial mRNA copy number and first-order degradation constants of mRNA. An appropriately trained MASK model calculated time-series gene expression profiles as accurately as a conventional kinetic model, in both a training and a test data set. The microarray data-based model also predicted expression profiles of yeast RP genes, controlled by multiple regulators, under various conditions. These validation results indicate that once a MASK model has been estimated from a microarray data set in which expression levels vary widely, that model is applicable to broad conditions. Thus, the MASK method will facilitate the prediction and elucidation of dynamic behaviours of genetic regulatory networks, which will be a major methodological advance in systems biology.

## Methods

### A rate equation for RNA synthesis

**Figure 4:**
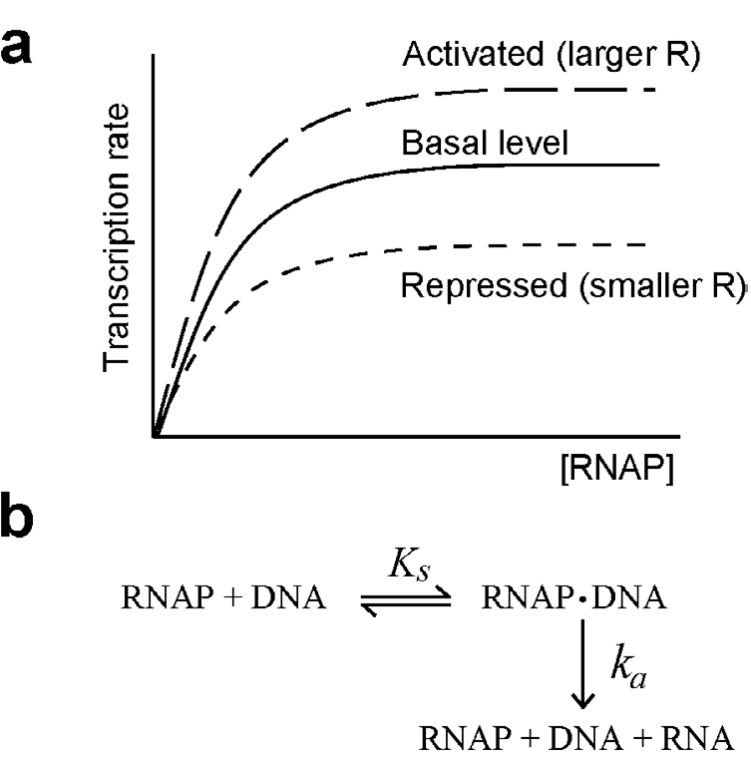
**A reaction mechanism postulated by Eq.(1). **(a) Variation of transcription rate defined by Eq,(1). Transcript rate is saturated as RNA polymerase increases. The R value determines maximum transcription rate. (b) A reaction scheme of RNA synthesis.

The RNA synthesis rate was assumed to be a product of the basal level transcription rate (L) and magnitude of regulation (R). The basal level transcription rate is hyperbolic with respect to RNA polymerase concentration (Figure 4a). The rate equation is as follows:

v=L⋅R=(ka[RNAP]KS+[RNAP])⋅R([Act],[Rep])     (1)
 MathType@MTEF@5@5@+=feaafiart1ev1aaatCvAUfKttLearuWrP9MDH5MBPbIqV92AaeXatLxBI9gBaebbnrfifHhDYfgasaacH8akY=wiFfYdH8Gipec8Eeeu0xXdbba9frFj0=OqFfea0dXdd9vqai=hGuQ8kuc9pgc9s8qqaq=dirpe0xb9q8qiLsFr0=vr0=vr0dc8meaabaqaciGacaGaaeqabaqabeGadaaakeaafaqadeGadaaabaGaemODayhabaGaeyypa0dabaGaemitaWKaeyyXICTaemOuaifabaaabaGaeyypa0dabaWaaeWaaeaadaWcaaqaaiabdUgaRnaaBaaaleaacqWGHbqyaeqaaOGaei4waSLaemOuaiLaemOta4KaemyqaeKaemiuaaLaeiyxa0fabaGaem4saS0aaSbaaSqaaiabdofatbqabaGccqGHRaWkcqGGBbWwcqWGsbGucqWGobGtcqWGbbqqcqWGqbaucqGGDbqxaaaacaGLOaGaayzkaaGaeyyXICTaemOuaiLaeiikaGIaei4waSLaemyqaeKaem4yamMaemiDaqNaeiyxa0LaeiilaWIaei4waSfcbiGae8NuaiLae8xzauMae8hCaaNaeiyxa0LaeiykaKcaaiaaxMaacaWLjaWaaeWaaeaacqaIXaqmaiaawIcacaGLPaaaaaa@6140@

where [RNAP], [Act] and [Rep] denote the concentration of RNA polymerase, activator and repressor, respectively. The kinetic parameters, *K*_*s *_and *k*_*a*_, represent the dissociation constant of RNA polymerase to DNA and the rate constant for RNA synthesis from the DNA-RNA polymerase complex, respectively (Figure 4b).

With respect to regulator genes, the R term is a function of their activators or repressors which should be described in terms of reaction kinetics. On the other hand, the R term of a target gene is defined as a power-law function of the R values of its regulators (Eq. (2)).

Rg(t)=a∏i=1nRi(t−τi)bi     (2)
 MathType@MTEF@5@5@+=feaafiart1ev1aaatCvAUfKttLearuWrP9MDH5MBPbIqV92AaeXatLxBI9gBaebbnrfifHhDYfgasaacH8akY=wiFfYdH8Gipec8Eeeu0xXdbba9frFj0=OqFfea0dXdd9vqai=hGuQ8kuc9pgc9s8qqaq=dirpe0xb9q8qiLsFr0=vr0=vr0dc8meaabaqaciGacaGaaeqabaqabeGadaaakeaacqWGsbGudaWgaaWcbaGaem4zaCgabeaakiabcIcaOiabdsha0jabcMcaPiabg2da9iabdggaHnaarahabaGaemOuai1aaSbaaSqaaiabdMgaPbqabaGccqGGOaakcqWG0baDcqGHsisliiaacqWFepaDdaWgaaWcbaGaemyAaKgabeaakiabcMcaPmaaCaaaleqabaGaemOyai2aaSbaaWqaaiabdMgaPbqabaaaaaWcbaGaemyAaKMaeyypa0JaeGymaedabaGaemOBa4ganiabg+GivdGccaWLjaGaaCzcamaabmaabaGaeGOmaidacaGLOaGaayzkaaaaaa@4CB7@

where *R*_*g*_(*t*) and *R*_*i*_(*t*-τ_*i*_) denotes the R value of a target gene *g *at time *t *and that of the *i*th regulator at time *t*-τ_*i*_, respectively. The term τ_*i *_represents time delay for transmitting regulatory effect of the *i*th regulator to the target gene *g*. The coefficients *a *and *b*_*i *_are parameters which can be estimated from microarray data. Regulatory effects at the translational level are abstracted by the coefficients of the R terms, such as exponential parameters and time delays. These equations were implemented on E-Cell Simulation Environment version 3.1.102 for Linux (Fedora Core 2/i386)[35].

### Parameter estimation from microarray data

**Figure 5:**
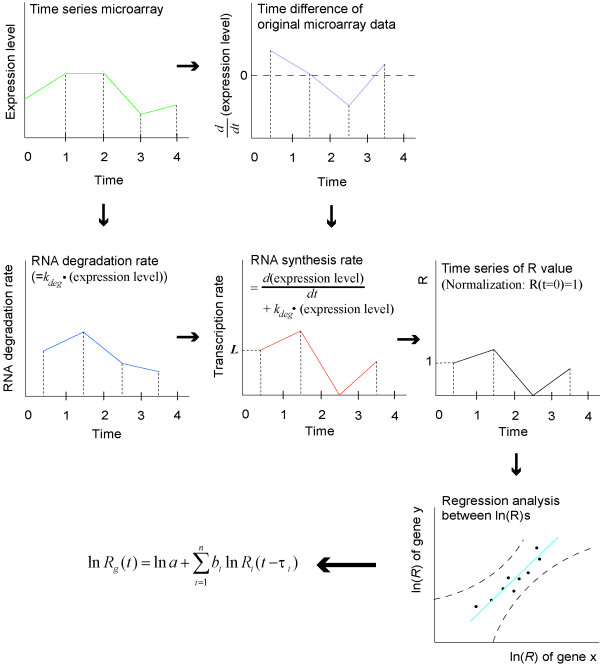
**Procedure for estimating the parameters of Eqs. (2) and (4). **The time derivative of an RNA level is the sum of the transcription rate and the degradation rate of RNA (top centre). The degradation rate is the product of the RNA level and the first-order degradation constant (centre left). Subtracting the degradation rate from the time derivative of microarray data, results in the RNA synthesis rate (centre). The time-series of R values are yielded by normalizing the synthesis rate as R(t = 0) = 1 (centre right). Eq. (3) is a mathematical representation of this procedure. Provided the time-series of the R values of regulators and target genes, the time delay τ_*i *_between the regulators and the target genes are calculated using the local clustering method[24]. Finally, a regression analysis of the time delay-corrected R time-series (bottom right) provides least-squares estimate of the coefficients in Eqs. (2) and (4) (bottom left).

A multiple regression analysis of time series of R values provides the coefficients in Eq. (2). A time-series of R values is obtained by following the data processing steps summarized in Figure 5: (i) differentiation of time-series microarray data with respect to time (ii) calculation of RNA degradation rate from first-order degradation constant (iii) summing up degradation rate and time derivative of expression level to obtain RNA synthesis rate (iv) normalizing RNA synthesis rate as rate = 1 at initial condition. Consequently, the R value at time t can be calculated by following Eq. (3) (See Additional Text 2 for derivation):

R(t)=Δarray(t)Δt+kdegarray(t)Δarray(0<t<1)Δt+kdegarray(0<t<1)     (3)
 MathType@MTEF@5@5@+=feaafiart1ev1aaatCvAUfKttLearuWrP9MDH5MBPbIqV92AaeXatLxBI9gBaebbnrfifHhDYfgasaacH8akY=wiFfYdH8Gipec8Eeeu0xXdbba9frFj0=OqFfea0dXdd9vqai=hGuQ8kuc9pgc9s8qqaq=dirpe0xb9q8qiLsFr0=vr0=vr0dc8meaabaqaciGacaGaaeqabaqabeGadaaakeaacqWGsbGucqGGOaakcqWG0baDcqGGPaqkcqGH9aqpdaWcaaqaamaalaaabaGaeuiLdqKaemyyaeMaemOCaiNaemOCaiNaemyyaeMaemyEaKNaeiikaGIaemiDaqNaeiykaKcabaGaeuiLdqKaemiDaqhaaiabgUcaRiabdUgaRnaaBaaaleaaieGacqWFKbazcqWFLbqzcqWFNbWzaeqaaOGaemyyaeMaemOCaiNaemOCaiNaemyyaeMaemyEaKNaeiikaGIaemiDaqNaeiykaKcabaWaaSaaaeaacqqHuoarcqWGHbqycqWGYbGCcqWGYbGCcqWGHbqycqWG5bqEcqGGOaakcqaIWaamcqGH8aapcqWG0baDcqGH8aapcqaIXaqmcqGGPaqkaeaacqqHuoarcqWG0baDaaGaey4kaSIaem4AaS2aaSbaaSqaaiab=rgaKjab=vgaLjab=DgaNbqabaGccqWGHbqycqWGYbGCcqWGYbGCcqWGHbqycqWG5bqEcqGGOaakcqaIWaamcqGH8aapcqWG0baDcqGH8aapcqaIXaqmcqGGPaqkaaGaaCzcaiaaxMaadaqadaqaaiabiodaZaGaayjkaiaawMcaaaaa@7B4A@

where *array *(*t*), Δarray(0<t<1)Δt
 MathType@MTEF@5@5@+=feaafiart1ev1aaatCvAUfKttLearuWrP9MDH5MBPbIqV92AaeXatLxBI9gBaebbnrfifHhDYfgasaacH8akY=wiFfYdH8Gipec8Eeeu0xXdbba9frFj0=OqFfea0dXdd9vqai=hGuQ8kuc9pgc9s8qqaq=dirpe0xb9q8qiLsFr0=vr0=vr0dc8meaabaqaciGacaGaaeqabaqabeGadaaakeaadaWcaaqaaiabfs5aejabdggaHjabdkhaYjabdkhaYjabdggaHjabdMha5jabcIcaOiabicdaWiabgYda8iabdsha0jabgYda8iabigdaXiabcMcaPaqaaiabfs5aejabdsha0baaaaa@3EEF@ and *k*_*deg *_denotes the relative expression level at time point t, the time difference of the first and second time points and the first order degradation constant of mRNA, respectively. Taking the natural logarithm of Eq. (2), we obtain,

ln⁡Rg(t)=ln⁡a+∑i=1nbiln⁡Ri(t−τi)     (4)
 MathType@MTEF@5@5@+=feaafiart1ev1aaatCvAUfKttLearuWrP9MDH5MBPbIqV92AaeXatLxBI9gBaebbnrfifHhDYfgasaacH8akY=wiFfYdH8Gipec8Eeeu0xXdbba9frFj0=OqFfea0dXdd9vqai=hGuQ8kuc9pgc9s8qqaq=dirpe0xb9q8qiLsFr0=vr0=vr0dc8meaabaqaciGacaGaaeqabaqabeGadaaakeaacyGGSbaBcqGGUbGBcqWGsbGudaWgaaWcbaGaem4zaCgabeaakiabcIcaOiabdsha0jabcMcaPiabg2da9iGbcYgaSjabc6gaUjabdggaHjabgUcaRmaaqahabaGaemOyai2aaSbaaSqaaiabdMgaPbqabaGccyGGSbaBcqGGUbGBcqWGsbGudaWgaaWcbaGaemyAaKgabeaakiabcIcaOiabdsha0jabgkHiTGGaaiab=r8a0naaBaaaleaacqWGPbqAaeqaaOGaeiykaKcaleaacqWGPbqAcqGH9aqpcqaIXaqmaeaacqWGUbGBa0GaeyyeIuoakiaaxMaacaWLjaWaaeWaaeaacqaI0aanaiaawIcacaGLPaaaaaa@55DC@

Note that multiple regression analysis of the time series of ln*R *yields an equation in the same form as Eq. (4). Thus, the least square estimates of ln *a *and *b*_*i *_were obtained via regression analysis of ln*R *time series data which are readily calculable from time series microarray data via Eq. (3). Target genes with regression p-values of more than 0.05 were not included in the mathematical models. The length of time delay, τ_*i*_, was calculated using the local clustering method [24].

### Estimation of *k*_*a *_from array data

The rate constant *k*_*a *_in Eq. (1) was determined for each gene to minimize mean relative error between experimental data and predictions. The mean relative error E of the two time series data sets was defined as follows:

E=1n∑i=1n|Xi−PiXi|
 MathType@MTEF@5@5@+=feaafiart1ev1aaatCvAUfKttLearuWrP9MDH5MBPbIqV92AaeXatLxBI9gBaebbnrfifHhDYfgasaacH8akY=wiFfYdH8Gipec8Eeeu0xXdbba9frFj0=OqFfea0dXdd9vqai=hGuQ8kuc9pgc9s8qqaq=dirpe0xb9q8qiLsFr0=vr0=vr0dc8meaabaqaciGacaGaaeqabaqabeGadaaakeaacqqGfbqrcqGH9aqpdaWcaaqaaiabigdaXaqaaiabd6gaUbaadaaeWbqaamaaemaabaWaaSaaaeaacqWGybawdaqhaaWcbaGaemyAaKgabaaaaOGaeyOeI0Iaemiuaa1aa0baaSqaaiabdMgaPbqaaaaaaOqaaiabdIfaynaaDaaaleaacqWGPbqAaeaaaaaaaaGccaGLhWUaayjcSdaaleaacqWGPbqAcqGH9aqpcqaIXaqmaeaacqWGUbGBa0GaeyyeIuoaaaa@4492@

where *X*_*i *_and *P*_*i *_denote the expression level at the *i*th time point of experimental data and predicted data, respectively. The symbol *n *represents the total number of time points. See Additional Text 3 for the detailed algorithm to calculate an optimal *k*_*a *_value for each gene.

## Authors' contributions

Yugi developed the mathematical aspects of MASK method and supervised the implementation of this method and the validation experiments. Nakayama provided the concept of the MASK method and directed the project. Kojima contributed to the development of simulation models for validation experiments. Kitayama implemented the method in the E-Cell system, and Tomita is a project leader.

## Supplementary Material

Additional File 1 **Detailed derivations and algorithms (Additional Text 1–3)**. A procedure to adjust R values for regulators, a derivation of Eq.(3) and an algorithm to calculate optimal *k*_*a *_are described in Additional Texts 1,2 and 3, respectively. Click here for file
