# Supplementary material for: A microarray data-based semi-kinetic method for predicting quantitative dynamics of genetic networks
Source: BMC Bioinformatics. 2005 Dec 13;6:299. doi: 10.1186/1471-2105-6-299 (PMC1326213; doi:10.1186/1471-2105-6-299)
Supplement: Additional File 1 — Detailed derivations and algorithms (Additional Text 1–3). A procedure to adjust R values for regulators, a derivation of Eq.(3) and an algorithm to calculate optimal ka are described in Additional Texts 1,2 and 3, respectively. [file 1471-2105-6-299-S1.doc]

**Additional file 1 of “A microarray data-based semi-kinetic method for predicting quantitative dynamics of genetic networks” (Yugi, K., Nakayama, Y., Kojima, S., Kitayama, T. and Tomita, M.)**

**Additional Text 1. A procedure to adjust R values for regulators**

Assuming that v_syn_ is constant between two adjacent time points, the mRNA concentration at the second time point (time t) is described as follows:

$[mRNA](t)=[mRNA](t=0)+v_{syn}\cdot t-\int_{0}^{t} k_{\text{deg}}[mRNA](\tau)d\tau$ (3.1)

Solving eqn.(3.1) in terms of *v_syn_*, we obtain,

$v_{syn}(t)=k_{\text{deg}}\frac{[mRNA](t)-[mRNA](0)e^{-k_{\text{deg}}t}}{1-e^{-k_{\text{deg}}t}}$ (3.2)

Since $v_{syn}=L\cdot R$, eqn.(3.2) yields,

$$R(t)=\frac{k_{\text{deg}}}{L}\cdot\frac{[mRNA](t)-[mRNA](0)e^{-k_{\text{deg}}t}}{1-e^{-k_{\text{deg}}t}}$$

**Additional Text 2. Derivation of Eq.(3)**

$\frac{d[mRNA](t)}{dt}=v_{syn}-k_{\text{deg}}[mRNA](t)$ (1.1)

$v_{syn}=L\cdot R$ (1.2)

Substitution of eqn. (1.2) with eqn. (1.1) yields,

$\begin{matrix} R(t) & = & \frac{v_{syn}}{L} \\ & = & \frac{1}{L}\left(\frac{d[mRNA](t)}{dt}+k_{\text{deg}}[mRNA](t)\right) \end{matrix}$ (1.3)

Normalizing R(t) to satisfy R(t=0)=1, we obtain

$\begin{matrix} v_{syn}(t=0) & = & L \\ & = & \frac{d[mRNA](0<t<1)}{dt}+k_{\text{deg}}[mRNA](0<t<1) \end{matrix}$ (1.4)

where [mRNA](0<t<1) denotes the mean of first and second time point of mRNA content.

Substituting eqn. (1.3) with eqn. (1.4), R(t) is represented below:

$R(t)=\frac{v_{syn}}{L}=\frac{\frac{d[mRNA](t)}{dt}+k_{\text{deg}}[mRNA](t)}{\frac{d[mRNA](0<t<1)}{dt}+k_{\text{deg}}[mRNA](0<t<1)}$ (1.5)

We assumed that each time point of a time series microarray data set represents an expression level relative to the first time point as indicated in eqn.(1.6) below:

$array(t)=\frac{[mRNA](t)}{[mRNA](0)}$ (1.6)

where array(t) denotes expression level (Cy3/Cy5 ratio) measured by microarray analysis.

Consequently, we obtain Eq.(3) from eqns. (1.5) and (1.6) as follows:

$$\begin{matrix} R(t) & = & \frac{[mRNA](0)\cdot\left(\frac{d}{dt}array(t)+k_{\text{deg}}array(t)\right)}{[mRNA](0)\cdot\left(\frac{d}{dt}array(0<t<1)+k_{\text{deg}}array(0<t<1)\right)} \\ & = & \frac{\frac{d}{dt}array(t)+k_{\text{deg}}array(t)}{\frac{d}{dt}array(0<t<1)+k_{\text{deg}}array(0<t<1)} \end{matrix}$$

Thus, it is possible to calculate R(t) from time series microarray data, initial copy number of RNA and the first-order degradation constant of RNA.

**Additional Text 3. An algorithm to calculate optimal *k_a_***

This Additonal Text explains an algorithm used to obtain the optimal value for *k_a_* which minimizes the mean relative error *E* with respect to the training data set.

$E=\frac{1}{n}\sum_{p=1}^{n} \frac{|[mRNA](p)_{ex\text{periment}}-[mRNA](p)_{prediction}|}{[mRNA](p)_{\text{experiment}}}$ (2.1)

We attempt to describe *E* as a function of *k_a_* prior to optimizing *k_a_* with respect to *E* Firstly, [mRNA](p) *_prediction_* is decomposed into *k_a_* and the other observable values.

$[mRNA](t=n+1)=[mRNA](t=n)+v_{syn}\cdot t-\int_{t=n}^{t=n+1} k_{\text{deg}}[mRNA](\tau)d\tau$ (2.2)

Solving eqn. (2.2), we obtain,

$[mRNA](t=n+1)=[mRNA](t=n)\cdot e^{-k_{\text{deg}}\tau}+\frac{v_{syn}}{k_{\text{deg}}}(1-e^{-k_{\text{deg}}\tau})$ (2.3)

Accordingly, the mRNA concentration at the *n*th time point can be represented by its initial concentration as below:

$[mRNA](t=n)=[mRNA](t=0)\cdot e^{-k_{\text{deg}}\sum_{i=1}^{n} \tau_{i}}+\sum_{j=1}^{n} \left\{\frac{e^{-k_{\text{deg}}\sum_{i=1}^{n} \tau_{i}}}{e^{-k_{\text{deg}}\sum_{m=1}^{j} \tau_{m}}}\cdot\frac{L\cdot R(t=j)}{k_{\text{deg}}}(1-e^{-k_{\text{deg}}\tau_{j}})\right\}$ (2.4)

Substitution of eqn. (2.4) into eqn. (2.1) yields,

$\begin{matrix} E & = & \frac{1}{n}\sum_{p=1}^{n} \frac{|[mRNA](p)_{ex\text{periment}}-[mRNA](p)_{prediction}|}{[mRNA](p)_{\text{experiment}}} \\ & = & \frac{1}{n}\sum_{p=1}^{n} \left|\frac{[mRNA](t=0)\cdot e^{-k_{\text{deg}}\sum_{i=1}^{n} \tau_{i}}}{[mRNA](p)_{\text{experiment}}}+\frac{k_{a}}{[mRNA](p)_{\text{experiment}}}\sum_{j=1}^{n} \left\{\frac{e^{-k_{\text{deg}}\sum_{i=1}^{n} \tau_{i}}}{e^{-k_{\text{deg}}\sum_{m=1}^{j} \tau_{m}}}\cdot\frac{L'\cdot R(t=p)}{k_{\text{deg}}}(1-e^{-k_{\text{deg}}\tau_{j}})\right\}-1\right| \\ & = & \frac{1}{n}\sum_{p=1}^{n} \left|a_{p}k_{a}-b_{p}\right| \end{matrix}$ (2.5)

The minimum value of *E=f*(*k_a_*) must be found in one of the $\frac{b_{p}}{a_{p}}$ values. Let $a_{p}^{'}$and $b_{p}^{'}$be the sorted series of $a_{p}$and $b_{p}$ in order of the smallest $\frac{b_{p}}{a_{p}}$ first. The optimal *k_a_* is,

$$k_{a}=\frac{b_{p}^{'}}{a_{p}^{'}}$$

where $\left\{\begin{matrix} 2S_{p}-S_{n}>0 \\ 2S_{p-1}-S_{n}<0 \end{matrix}\right.$ , $S_{k}=\sum_{p=1}^{k} a_{p}^{'}$
